# Supplementary material for: HIVprotI: an integrated web based platform for prediction and design of HIV proteins inhibitors
Source: J Cheminform. 2018 Mar 9;10:12. doi: 10.1186/s13321-018-0266-y (PMC5845081; doi:10.1186/s13321-018-0266-y)
Supplement: Supplementary file 2 — Additional file 2. Source code of HIVProtI web server. [file 13321_2018_266_MOESM2_ESM.zip › HIVprotI_Source-code/hivprotI/jsme/JSME_test.html]

JSME test page


Your web browser must have JavaScript enabled
in order for this application to display correctly.

# JSME test page

|  |  |
| --- | --- |
| Old look | New look |
| Turn on antialias | Turn off antialias |
| Turn on antialias for the molecular drawing area | Turn off antialias for the molecular drawing area |
| Drawing area line width: | set line width |
| Drawing area font size: | set font size |
| Drawing area scale: | set scale get scale |
| Non drawing area scale: | set scale get scale |

Turning AA on and off does not work with IE 9 & 10 (it is always on).

---

|  |  |
| --- | --- |
| Set options: | |
| reaction,noquery,oldlook,atommovebutton | Set options |
| Customize the help URL: http://peter-ertl.com/jsme/2013\_03/help.html | Set help URL |
| Display a message in the bottom bar: MW: XXXX.XXX | Show a message in the bottom bar |
| Customize the InChIKey search base URL: | Set InChIKey search base URL  | |
| Customize the InChIKey search using a custom function. In this example, the search function is replaced by opening a simple alert. | Set InChIKey search callback function |
| Reset InChIKey search to its default function. | Set InChi key search callback function to null |

|  |  |  |
| --- | --- | --- |
| Import predefined chemical structure to applet: | | |
| Read JME String | Read Multipart Structure | Read reaction |

|  |  |  |  |
| --- | --- | --- | --- |
| Export chemical structure from applet: | | |  |
| Get molfile | Get molfile V3000 | Get smiles | Get JME string |
|  | | |  |

|  |  |  |
| --- | --- | --- |
| Import chemical structure from text area above to applet: | | |
| Read MOL | Read JME String | Read any supported format |

## Known issues

IE9 64 bits in IE8 compatibility mode cannot display lines correctly
